# Supplementary material for: The effect of molecular assembly between collectors and inhibitors on the flotation of pyrite and talc
Source: R Soc Open Sci. 2019 Oct 9;6(10):191133. doi: 10.1098/rsos.191133 (PMC6837178; doi:10.1098/rsos.191133)
Supplement: Data [file rsos191133supp1.zip › data description.docx]

**Data description**

The main tool authors used in this study is origin. The detailed data and figures can be seen in the original data zip. Others not included in this zip were in the manuscript, such as Figure 2 and 10, because authors only have figure forms. Thank you for your understanding very much.
